# Macrocharcoal Signals in Histosols Reveal Wildfire History of Vast Western Siberian Forest-Peatland Complexes

**DOI:** 10.3390/plants11243478

**Published:** 2022-12-12

**Authors:** Viktor Startsev, Nikolay Gorbach, Anton Mazur, Anatoly Prokushkin, Lyudmila Karpenko, Alexey Dymov

**Affiliations:** 1 Institute of Biology of Komi Science Centre of the Ural Branch of the Russian Academy of Sciences, Syktyvkar 167982, Russia; 2 Institute of Natural Sciences, Pitirim Sorokin Syktyvkar State University, Syktyvkar 167000, Russia; 3 Center for Magnetic Resonance, St. Petersburg State University, University Av. 26, St. Petersburg 198504, Russia; 4 V.N. Sukachev Institute of Forest SB RAS, Krasnoyarsk 660036, Russia; 5 Department of Physics and Soil Reclamation, Faculty of Soil Science, Lomonosov Moscow State University, Moscow 119991, Russia

**Keywords:** boreal forest, charcoal, climate changing, peat soil, wildfires, ^13^C-NMR, PAHs

## Abstract

Fires are a naturally cyclical factor regulating ecosystems’ function and forming new postfire ecosystems. Peat soils are unique archives that store information about ecological and climatic changes and the history of past fires during the Holocene. The paper presents a reconstruction of the dynamics of fires in the subzone of the middle taiga of Western Siberia in the Holocene. Data on fires were obtained based on the results of a study of the content of macroscopic coal particles and radiocarbon dating. The effect of fires on soil organic matter (SOM) was estimated using ^13^C NMR spectroscopy and the content of polyaromatic hydrocarbons (PAHs). It is shown that throughout the Holocene, the peatlands studied were prone to fires. The conducted analyses show that the maximum content of charcoal particles is observed in the Atlantic (~9100–5800 cal. B.P.) and Subatlantic (~3100 cal. B.P. to the present) periods. The high correlation dependence of the content of coals with the content of PAHs (r = 0.56, *p* < 0.05) and aromatic structures of SOM (r = 0.61, *p* < 0.05) in peat horizons is shown, which can characterize these parameters as a reliable marker of pyrogenesis.

## 1. Introduction

Since the end of the last glacial period, high-latitude landscapes of the Northern Hemisphere have accumulated vast organic carbon pools in the peat soils. In the last decades, scientists have often identified variations in climate and the extreme rate of wildfires in the widespread territory of boreal forests [1,2]. Modern climate change, together with anthropogenic influence, is dangerous because it occurs quickly and in combination with many other irreversible changes in the Earth’s biosphere. However, in the geological time scale, climate change is not a new phenomenon. If we want to reveal how much climatic conditions change, it is worth paying attention to the natural climate variability during the Holocene (the last 11,800 cal. years BP). The most suitable is the Holocene climatic optimum, as it was the nearest long-term period (~10,000–5000 cal. years BP) when the average temperature exceeded present-day values [3].

Peatlands are important for the global carbon cycle, as they contain large reserves of carbon (C) in the biosphere [4]. Despite peatlands covering only 2–3% of the Earth’s land surface, they store about 25% of the carbon stored in global soils [5]. Deep and dense layers of organic material accumulate in peat bog ecosystems. In addition, peat soils are unique archives that store information about climatic and environmental changes; therefore, they are suitable for studying the history of wildfires in the past [6]. The study of fire history is possible due to the fact that post-pyrogenic carbonaceous particles are well preserved in peat [7,8]. The accumulation of charcoal occurs due to slow mineralization caused to chemical characteristics and the inability of decomposers to overcome oxygen deficiency conditions [9]. Sometimes it is possible to identify morphological features of coal particles in the fossil state in order to obtain information about the type of burned vegetation [10,11]. The increase in the content of coal particles in peat deposits can be considered an indicator of an increase in the number of fires associated with climate change and human activity [12].

In this study, we conducted a comprehensive assessment of peat soil formation in Western Siberia through pyrogenic history during the Holocene. The main idea was to trace the changes in the composition of peat and the chemical properties of peat soils over time to determine the markers of pyrogenesis. The aim of our study was to assess the impact of fires on the properties of histosols over the past 11,000 years. The main tasks were (i) to establish the stages of peat soil formation and peatland dynamics, (ii) to assess the content of charcoal particles in peat columns and fire ages, (iii) to identify the effect of fire on peat organic matter, and (iv) to determine the chemical markers of pyrogenesis.

## 2. Results

The studied soils, according to their morphological structure, belong to typical Histosols. Peat horizons are formed by plant reduces at different stages of decomposition (e.g., *Sphagnum*, *Carex spp.*, *Eriophorum sp*., pine etc.). Besides, the number of layers characterized by the presence of charcoals indicates the fire impact. The soils are strongly acidic (pH_KCl_ 2.7–3.9), characterized by a low base saturation (3–8%). The morphological and physicochemical characteristics of the studied peat soils are presented in more detail in [13].

### 2.1. Age Models and Chronologies

According to the results of radiocarbon dating (^14^C), the peat accumulation of studied peatlands (FH-1 and FH-2) started in the early (9.4 ka cal BP, 432 cm deep peat column) and middle Holocene (8.4 ka cal BP, 260 cm deep peat column), respectively (Table 1).

**Figure 1:**
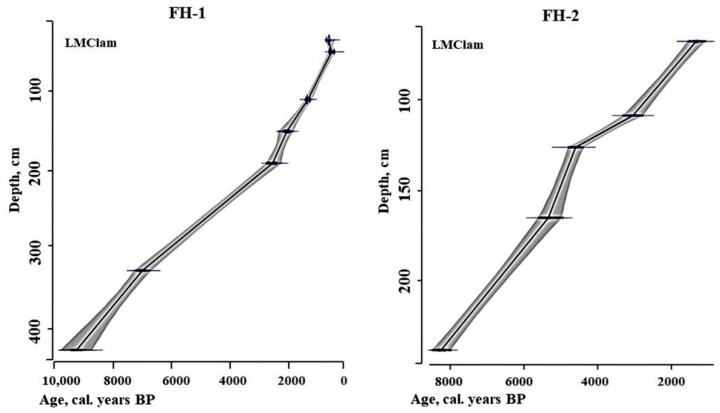
Model of the rate of vertical growth of studied peats (depth to age).

A vertical growth rate of peat was constructed due to the model shown in Figure 1. The peat growth rate of the FH-1 site in the Boreal period of the Holocene (9350–7100 cal. years BP) was 0.5 mm/year. For a long period of 7100–2600 cal. years BP, peat growth was 0.3 mm/year. The maximum peat growth rate (up to 1 mm/year) was found for the period 2600–2160. In the periods of 2160–1400 and 1400–500 cal. years BP, the growth rate was 0.5 and 0.7 mm/year. However, in the last 500 years, the growth rate of peat has increased again to 1 mm/year.

Peat accumulation in FH-2 demonstrated the lowest rate of growth (0.3 mm/year) at an early stage of development (8380–5500 cal. years BP). In the period from 5500 to 4700 cal. years BP, the growth rate increased to 0.5 mm/year. In the period of ~4700–3100 cal. BP, the rate drops to 0.1 mm/year and then begins to increase sequentially. In the period of 3100–1100 cal. years BP, the average growth rate was characterized by values up to 0.21 mm/year. The maximum growth rate was detected in the period from 1100 cal. years BP to the present–0.6 mm/year.

### 2.2. The Botanical Composition of Peat

**Figure 2:**
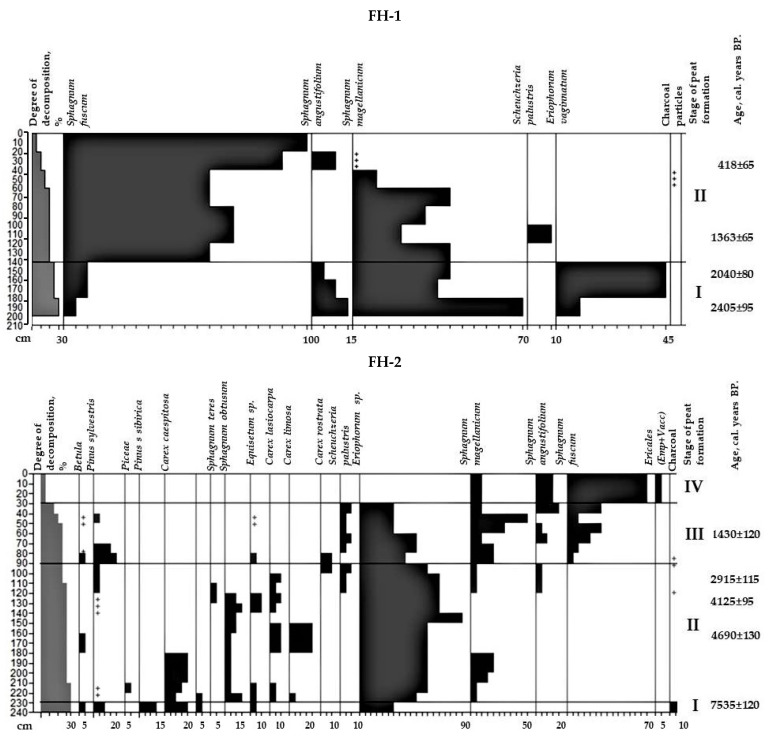
Diagram of the botanical composition of studied peats. I–IV—stages of peat formation.

The analysis of the botanical composition of peat is presented in the diagram in Figure 2. The peat development in the studied area FH-1 included two stages. The initial stage of peat accumulation falls on a segment of 9300 cal. years BP. Presumably, the formation of the swamp began with the overgrowth of shallow ponds with hydrophilic plants. The next mesotrophic phase was represented by transitional sphagnum peat (180–200 cm). Oligotrophic plant associations were established at the border (transition) from the Subboreal to Subatlantic periods (~2500–3000 cal. BP) and formed a significant mass of peat (180–0 cm).

The studied FH-2 peatland has also passed through several stages in its development. Its formation began with the mesotrophic phase around 8000 years ago at the transition of the Boreal and Atlantic periods of the Holocene with the waterlogged forested areas. This is evidenced by the presence of wood species residues (30%) in the peat bottom. This type of peatland is characterized by a small area of pine-shrub-sphagnum peat.

### 2.3. Charcoal Particles

**Figure 3:**
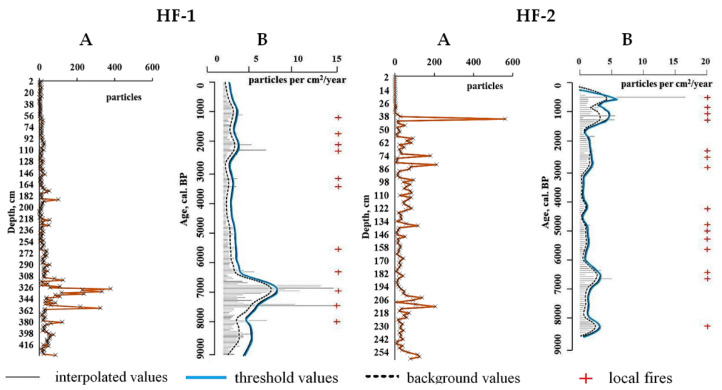
The amount and rate of accumulation of charcoals in the studied peat. (**A**) is the amount of coals in the soil profile (particle) and (**B**) is the model of the rate of coal accumulation (particles per cm^3^/year).

The presence of charcoal particles in studied peat columns evidences the regular appearance of fires in the study area during the Holocene The peat bog FH-1 was exposed to 11 local fires (Figure 3). The number of charcoal particles found during the Boreal period was 918 particles. It was found that five fires took place in the Atlantic period from 8000 to 5000 cal. years BP. During this period, 3571 pcs. charcoal were detected. The highest concentrations of charcoal particles in the peat FH-2 are observed in the period ~ 7000–6200 cal. years BP. (108–378 pcs.). The maximum coal content was detected at a depth of 327 cm (378 pcs.) and dates back to ~6759 cal. years BP. In the Subboreal period, there was lower fire activity. During this period, 551 pcs. coal were found. Two local fires were recorded in the period ~3500–3200 cal. years BP. (146 pcs.). High fire activity was noted in the Subatlantic period, in the time interval of ~1000–2500 cal. years BP. During this period, the presence of 1215 pcs. charcoal particles was detected, and four local fires were established.

The peat bog FH-2 experienced 15 local fires during the Holocene. In total, there were 5592 pcs. charcoal found in the studied peat bog in the entire column. In the lower part of the profile, which belongs to the early Atlantic period of the Holocene (~9000–8000 cal. years BP), 598 charcoal particles were found in the studied peat soil. In addition, one local fire was detected, which is confirmed by the presence of layers with increased charcoal content (116–126 particles). Seven local fires were recorded during the Atlantic period (~8000–5000 cal. BP.) and the beginning of the Subboreal period (~5000–4000 cal. years BP). We detected 2101 pcs. charcoal in the peat column layer dated to this period. The maximum values ranged from 105 to 206 pcs. (~4275–4096 and ~6646–6364 cal. years BP.). A sharp increase in the rate of accumulation of charcoal particles at the end of the Holocene was noted, and seven local fires have been established since the end of the Subboreal period (~3000–500 cal. years BP.) and the Subatlantic period to the present (~1600–500 cal. years BP.). It was revealed that 2893 pcs. charcoal were accumulated during this period. The highest rate of coal accumulation was observed at a depth of 25 cm and dates back to the end of the Subatlantic period (~428 cal. years BP.). This layer contains the maximum content of charcoal particles (562 pcs.).

### 2.4. Carbon and Nitrogen Contents

The studied peat soils are characterized by high carbon content (Table 2). The carbon content in soil FH-1 ranged from 456 to 563 g kg^−1^. The nitrogen content in the peat FH-1 varied from 6.7 to 36 g kg^−1^. In soil FH-2, the carbon content in peat horizons varied from 258 to 535 g kg^−1^. The mineral horizon G (27 g kg^−1^) is characterized by the lowest carbon content. Soil FH-2 contains from 7.1 to 31 g kg^−1^ of nitrogen.

As a result of the analysis of water-soluble organic compounds, the maximum concentrations were found for the middle part of the peat profile (40–140 cm). The distribution of WSOC and WSON content was uneven across the FH-1 peat profile. The FH-1 peat contained 1.36–2.15 mg g^−1^ of water-soluble organic carbon and 0.05–0.07 mg g^−1^ of water-soluble organic nitrogen. The maximum content was detected at depths of 60–80 cm. The content of water-soluble carbon WSOC in the peat soil FH-2 profile varied from 0.09 to 1.63 mg g^−1^, and the concentration of nitrogen WSON varied from 0.03 to 0.17 mg g^−1^. There was a gradual decrease in the WSOC content from a depth of 180 cm (1.21 mg g^−1^) to the mineral horizon (0.09 mg g^−1^). The maximum content of 1.63 mg g^−1^ was detected at a depth of 120–140 cm.

The analysis of stable isotopes showed that the values of δ ^13^C varied in the peat FH-1 from −28.21‰ to −25.54‰ (−27.08 ± 0.43) and δ^15^N varied from −2.35 to +1.63‰. The isotopic composition of peat FH-2 is characterized by higher values δ ^13^C from –28.04‰ to −24.07‰ and δ^15^N from −4.6 to +1.51‰. The calculation of carbon (C_stock_) and nitrogen (N_stock_) stocks allowed us to establish that peat FH-1 contains 149.7 kg m^–2^ of carbon and 4.7 kg m^–2^ of nitrogen. Peat FH-2 contains 184.1 kg m^–2^ of carbon and 7.8 kg m^–2^ of nitrogen.

### 2.5. CPMAS ^13^C NMR Data

The results of ^13^C-NMR spectroscopy on the studied peats are presented in Table 3. It was found that aliphatic fragments predominate in the studied peats (67–87%). The composition of organic matter in the peat FH-1 is not homogeneous. The composition of SOM in the upper part of peat (0–200 cm) is dominated by aliphatic fragments (81–87%). Their composition is dominated by alkyl-C fragments (15.4–25.9%) and groups associated with hemicellulose C_Alk-O_ (35.9–51.2%) and cellulose C_O-Alk-O_ (9.9–13.3%). Fragments of aromatic structures of Aryl-C constitute 13–19%. Carboxyl groups (Carboxyl-C) account for 3–4% of the composition of the peat organic matter shifts from a depth of 200 cm. There is a gradual decrease in the proportion of fragments associated with cellulose-like substances (6.0–8.4%) and hemicellulose (23.2–32.5%) in the soil profile. The proportion of methoxyl C_CH3-O_ (7.1–9.5%) and carboxyl (7–10%) groups and alkyl-C fragments (21.6–32.7%) increases. The proportion of aromatic fragments increased in the lower part of the soil profile (up to 27%). The ratio of alkyl/O,N-alkyl demonstrates the downward increase in profile, reflecting the stronger degree of decomposition of peat organic matter. This increase is most pronounced from 200 cm depth.

The composition of SOM at a depth of 20–230 cm is quite stable. Aliphatic fragments in peat also predominate throughout the peat profile of FH-2 peat bog (up to 84%). The largest share falls on the upper part of the profile, with a predominance of O-alkyl groups (up to 61%). The proportion of aromatic fragments in the SOM composition is about 16–23% to a depth of 215 cm and increases to 33% in the pyrogenic horizon T_pyr_ (235–240 cm). In the composition of aliphatic fragments, the main share is accounted for by C_Alk-O_ (12.6–41.1%) and alkyl-C fragments (16.2–37.6%). Compounds associated with cellulose-like substances C_O-Alk-O_ (5.3–12.2%) and C_CH3-O_ methoxyl groups (6.8–11.5%) occupy smaller shares. The degree of decomposition of organic matter, as shown by the fact that the ratio of alkyl/O,N-alkyl gradually increases along the soil profile from 0.3 to 1.5.

The upper peat layer and the pyrogenic horizon T_pyr_ of the FH-2 profile differ significantly from the main peat mass. The horizon T (0–20 cm) is represented by the least decomposed plant residues and is characterized by a minimum ratio of alkyl/O,N-alkyl (0.3). It is dominated by O-alkyl fragments (61%) associated with cellulose-like (12.2%) and hemicellulose substances (41.4%). There is also an increased content of aromatic components due to substituted C_Ar-O,N_ (6.2%). Pyrogenic horizon Tpyr contains an elevated portion of aromatic fragments (33%), mainly due to fragments C_Ar-H(C)_ (26.4%). The horizon is characterized by a minimum content of fragments of hemicellulose (12.6%), cellulose (5.3%) and methoxyl groups (6.8%). The organic matter of the pyrogenic horizon T_pyr_ is maximally decomposed in the soil profile according to the ratio alkyl/O,N-alkyl (1.5).

### 2.6. Concentration and Distribution of PAHs

**Figure 4:**
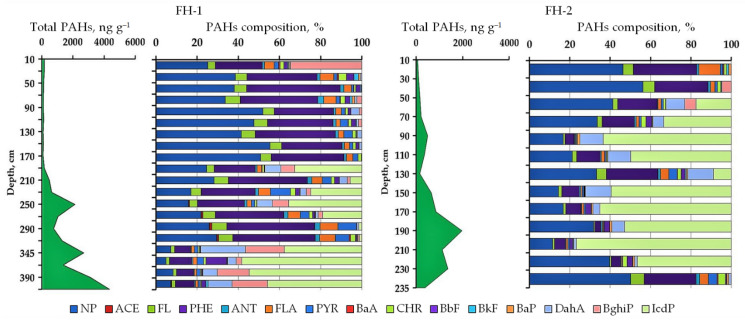
The profile distribution and composition of PAHs in studied histosols. NP–naphthalene, ACE–acenaphthene, FL–fluorene, PHE–phenanthrene, ANT–anthracene, FLA–fluoranthene, PYR–pyrene, BaA–benzo[a]anthracene, CHR–chrysene, BbF–benzo[b]fluoranthene, BkF–benzo[k]fluoranthene, BaP–benzo[a]pyrene, DahA–dibenzo[a,h]anthracene, BghiP–benzo[g,h,i]perylene, IcdP–indeno[1,2,3-c,d]pyrene.

The content of PAHs in the studied peats varied from 0 to 2000 ng g^−1^ in soil FH-1 (Figure 4, Appendix A Table A1). Peat FH-2 contained from 0 to 1043 ng g^−1^ PAHs. The predominant PAHs in the peat are 2–3-4 nuclear compounds (naphthalene, phenanthrene, fluoranthrene), 5-nuclear dibenzanthracene, and 6-nuclear indenopyrene. The nature of the PAH distribution in peat profile HF-1 is uneven. The composition of PAHs of the upper 200 cm is dominated by light compounds. The content of naphthalene was 42–64 ng g^−1^, phenanthrene–32–59 ng g^−1^. The remaining connections were much smaller. The total PAH content in the upper part of the profile was 99–173 ng g^−1^. Peat HF-1 is characterized by a sharp increase in the content of all PAHs from a depth of 200 cm. The maximum content was found for naphthalene (up to 390 ng g^−1^), phenanthrene (up to 530 ng g^−1^), dibenzanthracene (up to 590 ng g^−1^), benzoperylene (up to 510 ng g^−1^) and idenopyrene (up to 2000 ng g^−1^). The total PAH content in peat HF-1 increases with depth (99–4370 ng g^−1^). Up to a depth of 330 cm, light compounds of 2-3-4-nuclear PAHs (99–1282 ng g^−1^) predominate in the composition of PAHs, deeper there is an increase in the content of 5–6-nuclear compounds (up to 3404 ng g^−1^).

The PAH content in peat FH-2 gradually increased down the profile to a depth of 200 cm. After that, there is a gradual decrease in the PAH content. The total content varies from 71 to 1974 ng g^−1^: naphthalene (33–627 ng g^−1^), phenanthrene (22–99 ng g^−1^), dibenzo[a,h]anthracene (0–126 ng g^−1^) and idenopyrene (0–1043 ng g^−1^) were characterized by a predominant content of PAHs. It was found that the concentration of high-molecular 6-nuclear dibenzo[a,h]anthracene prevailed from a depth of 100 cm, except for the lower peat horizon (0 ng g^−1^), in which light PAHs predominated. An increase in the content of naphthalene (192–545 ng g^−1^) was also detected in the pyrogenic and over-pyrogenic layers.

## 3. Discussion

### 3.1. Peat Formation in the Context of Pyrogen History

The paludification process and the accumulation of peat in our study territory began at 14,700–12,700 cal. years BP [14]. The research area is unique in terms of coverage of the processes of peat bog formation and peat accumulation. The vegetation of the studied territory is represented by communities of wetland pine forests. The average waterlogging of the study territory was about 50%; in some areas, it reached 70–75% [15]. Pine sphagnum and shrub-sphagnum forests replace each other, which are then replaced by pine shrub-sphagnum forested peat bogs [16,17]. It was revealed that the studied peatlands differ in the rate of vertical growth in certain periods of the Holocene. These differences reflect the development of the studied peatlands at different stages of the Holocene and their current state. At the same time, it is important to take into account that the rate of vertical growth in boreal ecosystems is due to a variety of natural factors.

Considering the botanical composition of the peat FH-1, it can be argued that at first, a sedge-sphagnum phytocenosis was formed with the dominance of *Eriophorum* on a cover of *Sphagnum magellanicum* with an admixture of *S. angustifolium*. Then a sphagnum community with the dominance of *S. fuscum* emerged. *Fuscum* peat contains the remains of hydrophilic *Scheuchzeria palustris*. During the upper stage of the development of the peat, which began to be established at the border Subboreal and Subatlantic periods and continues to the present, the forest areas surrounding the swamp were regularly exposed to fires [2,18]. This is indicated by the presence of charcoal particles diagnosed in all layers of peat. However, their small number and the absence of pronounced pyrogenic layers serve as indirect evidence that fires did not affect the watered part of the peat, and their intensity was not high.

At the initial stage (I) of development, peat FH-2 was affected by a fire (7535 ± 120 cal. BP). This is confirmed by the presence of burnt bark and wood residues in the botanical composition of peat (charcoals 10%). The remaining peat-forming plants have retained their anatomical structure. The peat is dominated by the remains of *Betula sp*., *Pinus sibirica*, *P. sylvestris* (5–15%), *Equisetum*, *Carex caespitosa* and *Eriophorum* (55% in total). At the next mesotrophic stage (II), covering the entire Atlantic and Subboreal period, paleophytocenoses are formed with the dominance of *Eriophorum* (50–90%) in the botanical composition of peat (230 to 90 cm). In addition, the samples contain the remains of woody plants (*Betula*, *Pinus*, *Picea*), sedges (*Carex caespitosa*, *Carex lasiocarpa*, *C. limosa*), *Equisetum* and *Sphagnum* mosses (*Sphagnum obtusum*, *S. teres* and *S. magellanicum*). Communities existed throughout the Atlantic and Subboreal periods of peat development (8050–2915 cal. BP). The presence of charcoal particles serves as evidence that the adjacent forest territories regularly experienced the burning. At the same time, the studied swamp was not affected by fires due to its high water content, as evidenced by the abundance of hydrophilic plants in the composition of phytocenoses. The absence of large fires is indirectly confirmed by the high degree of preservation of peat-forming plants. At the end of the Subboreal and the beginning of the Subatlantic period, the swamp passed to a new stage of development (III-IV). Successions occurred gradually without a sharp change in humidification conditions. Mesotrophic *Carex lasiocarpa*, *C. caespitosa*, *Sphagnum obtusum*, *S. teres* and others disappeared from the associations. They were replaced by oligotrophic *Scheuchzeria palustris*, *Sphagnum fuscum* and *S. angustifolium*. At the final stage, marsh shrubs (*Ledum palustre*, *Andromeda polifolia*, *Chamaedaphne calyculata*, etc.) began to appear. As a result, a shrubby-sphagnum swamp was formed, similar in composition and structure to the one currently existing. In the sub-Atlantic period (approximately the last 2500 years), the impact of fires and adjacent forests on the swamp was regular.

The data obtained by us are consistent with the results of L.V. Karpenko [18,19] for this region (11,800–10,200 cal. BP) and the central part of the West Siberian lowland (9000–11,500 cal. years BP) [20]. According to the authors [14], the differences can be explained by the methods of determining the age and the calibration parameters of the method. Nevertheless, in most studies, approximately the same age of peat accumulation in swamp ecosystems, for example, in Western Siberia (8500 cal. BP) [21] and the European North of Russia (11,010 cal. BP), in the Holocene [7,22] was observed. All researchers agree that fires affect the development of peat soils in the boreal zone. The pyrogenic features of peatlands reflect the modern development of bogs and their individual stages [2]. Knowledge about the rate of carbon accumulation and its changes over time is an important step in modern research. According to [23,24], peat and tundra ecosystems are able to shift from a carbon sink into its source due to increased soil respiration under warmer and drier conditions. Therefore, in order to better understand current and future ecosystem pyrogenic changes, it is necessary to study and understand their relationship with vegetation and climatic changes in the past [25].

### 3.2. Charcoal Particles as a Marker of Pyrogenesis

Charcoal particles are present throughout both analyzed peat soil profiles. This indicates that fires were constantly occurring during all periods of the Holocene when the studied peatlands were being developed. Probably, the presence of charcoals reflects the natural causes of fires associated with climatic conditions in Western Siberia [26]. Differences in the content of coal particles were revealed between the studied peat soils. Peat bog FH-2, in comparison with peat bog FH-1, is characterized by an increased frequency of local fires. This is probably due to the fact that the peat FH-2 has a smaller area and is surrounded by forest, which has been exposed to fires more often. The FH-1 peat bog developed in an open area and at a distance from the forest and was less often subjected to pyrogenic influence. In contrast, the peat bog FH-1 is characterized by a higher concentration of pyrogenic coals in general. This may indicate a higher intensity of fires or the ability to capture more charcoals. The increased content of pyrogenic coals was found in the deepest horizons of the studied peatlands. This indicates fires (or a series of fires) could trigger the formation of peats in this area.

The longest period of time with a high rate of coal accumulation occurred during the Atlantic period. According to the literature [27,28,29,30,31,32], in the Atlantic period, the temperature exceeded the current values by 3.4–5 °C, which probably affected the increase in the frequency of fires. Similar data are presented in the works of other authors [21,33]. It was also revealed that the summer values of solar radiation in the boreal zone reached their maximum values during the Holocene optimum (~9000–5000 cal. years BP), and, probably, this led to an increase in fires. The dynamics of fires could also be influenced by the taxonomic composition of plants in the Boreal biome in the Holocene [34]. Probably, plant communities resistant to fires were replaced by fire-hazardous ones. During the Subboreal and the beginning of the Subatlantic periods, the proportion of fire-hazardous plants was less, which led to a lower rate of accumulation of pyrogenic plant residues.

Thus, with an increase in summer air temperature, a decrease in climate moisture and a change in plant communities, we can expect an increase in the number and scale of fires in the middle taiga of Western Siberia. Both peatlands are characterized by an increase in the rate of charcoal accumulation at the end of the Holocene. Similar results were published in works on the European part of Russia [8,35], where the authors associate an increase in the rate of charcoal accumulation at the end of the Holocene with an increase in anthropogenic influence. A similar increase in the frequency of fires over the past two millennia has also been recorded in southern Siberia, such as Lake Baikal [25] and in the Altai Mountains [36,37].

### 3.3. Fire Impact on Soil Organic Matter

Knowledge about the rate of carbon accumulation and its changes over time is an important step in modern research of soil organic matter. According to [23,24,38], peat ecosystems from a pure carbon sink are able to turn into a source due to increased soil respiration under warmer and drier conditions. The high content of carbon, nitrogen and their water-soluble fraction is typical for peatlands, which is consistent with the data on Western Siberia, Finland and Canada (420–630 g kg^−1^) [39,40,41,42]. The results of this study are comparable with those previously published for the study region (460–570 g kg^−1^) [43]. The studied peats largely depend on peat-forming plants in terms of carbon and nitrogen content. Peat from vascular plants is characterized by a higher content of C and N than from sphagnum [43,44,45]. So, for peat FH-1, the upper part (0–180 cm) is characterized by lower concentrations of carbon and nitrogen than in deeper layers. There is a sharp transition to an increase in the carbon content in the deeper layers of peat (180–420 cm). This indicates differences in the botanical composition of the peat. Similar regularities were revealed for peat FH-2, where vascular plants (*Scheuchzeria*, sedge, etc.) increased in the depth of the profile in the composition of peat. The nitrogen concentration also increases with depth as the degree of decomposition increases. At the same time, the C/N ratio narrows. The C/N ratio in the peat FH-1 was 14–95. In the peat FH-2, the ratio varied from 20–79. The highest values of the C/N ratio are characteristic of the upper peat horizons. There is a noticeable transition from the sphagnum type of peat with a low nitrogen content to the type of peat with more significant nitrogen concentrations, which is probably due to the predominance of vascular plants in the deep layers of peat [19]. In general, the nitrogen content in the peat of the temperate and boreal zones varies between 4.0–29 g kg^−1^ [44]. There is no obvious effect of pyrogenesis on the carbon and nitrogen content. However, we assume that the impact of wildfire may affect carbon and nitrogen stocks.

In terms of peat reserves, Russia ranks first in the world with 3.691 million km^2^ or 21% of the country’s territory. The world reserves of carbon accumulated in swamps (in an area of 6.41 million km^2^) amount to 329.0–528.0 Gt of carbon [46]. Significant differences in carbon reserves in the studied soils can be explained by the density of peat. The peat density of the HF-2 site is significantly higher than that of the FH-1 site. The lower bulk density of the soil FH-1 is explained by the higher rate of accumulation of young sphagnum mosses [47,48]. At the FH-1 site, the sphagnum gained not only height but also carbon faster compared to the FH-2 soil. The influence of fires on soil density should not be excluded. The works [45] show an increase in density after fires due to the accumulation of ash. The impact of fires may also be reflected in an increase in pyrogenic carbon (PyC) content [2]. More frequent fires at the FH-2 site are also likely to affect the increase in carbon stocks.

The molecular composition of peat and the degree of its humification is determined by the geographical location, vegetation and environmental conditions. The most common components in the organic matter of peat soils include cellulose, hemicellulose and lignin, which are mainly derived from plants [49]. Thus, peat is very heterogeneous and rich in organic carbon material.

The molecular composition of the studied peat is characterized by the predominance of aliphatic fragments. As is known, pyrogenesis is an important factor that determines the content of aromatic fragments in the composition of organic matter [50,51]. Horizons with clear signs of fire impact at depths of 219, 331, 399 and 413 cm are distinguished in the soil of FH-1. The contents of aromatic fragments in them are 20%, 27%, 26% and 27%, respectively. The maximum values of the degree of aromaticity (AR/AL) were found in the same horizons. Also, in these horizons, as the proportion of aromatic fragments increases, an increased content of coal particles is revealed. It can be assumed that fires led to an increase in the aromaticity of organic matter. The correlation coefficient between the content of coals and CAr-H(C) is significant (r = 0.63, *p* < 0.05) and for CAr-O,N (r = 0.56, *p* < 0.05) and for the general degree of aromaticity (fa; r = 0.61, *p* < 0.05). Data on molecular fragments in the organic composition of soil FH-2 showed an increase in aromatic fragments down the profile. The lower peat horizons stand out especially, one of which is pyrogenic. As it was noted earlier, the studied peat bog was exposed to fire at the beginning of its formation. This explains the significant increase in Aryl C and the overall aromaticity of organic matter. Fires and pyrogenic residues (charcoal particles) are probably the reason for the high aromaticity of peat horizons of the studied soils. The preservation or retarded decomposition of these residues was facilitated by the fact that they are least actively processed by destructors [9,52,53].

### 3.4. Isotopic Composition and PAH Content as a Criterion for Assessing the Impact of Fire

The stable isotope composition of soils can be considered a criterion for detecting environmental changes. This is especially true in peat soils, which are important archives containing information about the history of peat formation, climate change and past fires [6,54,55].

Peat consists of various plant residues that have undergone transformations as a result of the activity of microorganisms, and, first of all, the isotopic composition is influenced by peat-forming plants. In the botanical composition of peat FH-1, *Sphagnum spp*. dominated in the upper two meters. The values of δ^13^C in peat FH-1 ranged from −27.84 to −26.57 to a depth of 180 cm after δ^13^C began to increase (−26.66‰–−25.54‰). Deeper peat horizons (220–420 cm) are characterized by a decrease of δ^13^C values from –26.95‰to –28.03‰. An increase in the isotopic composition may also indicate a change in the hydrological regime towards a decrease in the humidity of peat horizons. An increase in δ^13^C with a depth in sphagnum peat in the upper 100 cm suggests wetter conditions during the transition to another stage of peat development [43]. In peat FH-2, the δ^13^C values increased to −25.31%–−24.07‰ up to a depth of 120 cm, followed by a decrease in deeper horizons (−25.14‰…−28.04‰). In this peat at depths from 100 to 240 cm in the botanical composition, there was a change in the dominant peat-forming plants; namely, the *Sphagnum* disappeared, and *Eriophorum* appeared. In the mesotrophic stage of peat bog formation, fluffy phytocenoses prevailed, which deposited the bulk of peat (50–90%).

The δ^15^N values are characterized by similar patterns and are within the range of reported values for peat bogs from the Subarctic and Arctic Regions [56,57]. For the peat ecosystems considered, there is an obvious period of increase in total N and δ^15^N with depth. This period is characterized by the dominance of Eriophorum. This is consistent with the data of [58], where the predominance of *Scheuchzeria palustris L*. was noted (up to 50–70%). Similar results were obtained by the authors when studying different peatlands [59,60,61]. The characterization of the isotopic composition allows us to assess changes in moisture conditions, the type of peat vegetation and the degree of decomposition of organic matter. Nevertheless, we found a negative correlation coefficient between the δ^13^C and the charcoal content in the peat profile FH-1 (r= −0.47, *p* < 0.05). The negative correlation of δ^13^C values is shown with the aromatic structures of SOM (r= −0.53, *p* < 0.05). In contrast, δ^13^C has a positive relationship with aliphatic fragments of SOM (r = 0.53, *p* < 0.05).

It Is difficult to assess the direct impact of fires on δ^13^C and δ^15^N of peat. The revealed correlations should be discussed from the standpoint of the transformation of plant residues and the formation of peat by different types of peat-forming plants, which more clearly reflect the content of the isotopic composition. However, some authors suggest that fires lead to an increase in δ^13^C values [59]. Polyaromatic hydrocarbons are high-molecular structures, the sources of which can be both natural and man-made processes. The natural sources of PAHs in peat soils are usually attributed to the intake from rocks and oils [62], vegetation [59,63,64] and fires [2,65,66]. The content of PAHs in the studied peat soils is characterized by a tendency to increase the content of polyarenes deep into the profile. The predominance of light PAHs was revealed (Figure 4, Table A1). Their share in peat FH-1 ranges from 1 to 52%. However, after 180 cm, there is an increase in the content of heavy PAHs, in particular, indeno[1,2,3-c,d]pyrene. The proportion of heavy PAHs after it varied from 1 to 58%. Similar patterns have been identified in peat FH-2. The prevalence of heavy PAHs after 140 cm was up to 65%.

The features of PAH distribution are the result of the influence of various factors-botanical composition, redistribution of substances in peat, the degree of decomposition of SOM and pyrogenesis. Fires lead to an increase in the PAH content in soils [1,2]. Some authors note that the sources of PAHs are not only modern fires but also fires that have passed a long time ago (paleofires) [65,67]. In our work, we revealed an increase in the content of PAHs in horizons with pyrogenic features. High correlation values were obtained between the content of PAHs and the content of coal particles in peat horizons. This is especially clear for soil FH-1. The correlation coefficient between ∑PAH and the content of coal particles was r = 0.56, *p* < 0.05. Several individual PAHs with significant correlation coefficients with the charcoal contents were revealed: naphthalene (r = 0.53, *p* < 0.05), chrysene (r = 0.65, *p* < 0.05), dibenzo[a,h]anthracene (r = 0.70, *p* < 0.05). Presumably, coal particles formed as a result of fires (paleofires) can participate in the formation of PAHs. At the same time, significant concentrations of high-molecular PAHs are associated with their content in certain plant species. Deep peat horizons are characterized by an increase in herbaceous vegetation containing most of the lignin. Lignin is the main source of organic matter of no-pyrogenic origin [68]. In addition, the PAH content significantly correlates with aromatic fragments (markers of pyrogenesis) of organic matter (r = 0.77–0.78, *p* < 0.05) and negatively with aliphatic fragments (–0.78, *p* < 0.05). In [67], it was noted that pyrogenesis is a key factor in the formation of PAHs, and most of them are pyrogenic. The most frequently encountered PAH ratios in the literature were calculated to assess whether the isolated PAHs are pyrogenic [69,70,71,72]. The calculated coefficients are presented in Table A2.

The ratios ANT/178, ANT/ANT+PHE, FL/PYR and PYR+BaP/CHR+PHE showed borderline values of PAH origin. This indicates that the observed PAHs have a mixed origin from oil and from fires. In the studied peats, the ANT/178 and ANT/ANT+PHE ratios show that PAHs in the upper part of the soil profile (up to the “turning point”) do not have pyrogenic origins. From the depth (200 cm for peat FH-1 and 120 cm for peat FH-2), boundary values (>0.1) were detected. This may indicate the involvement of pyrogenesis in their formation. The ratio PYR+BaP/CHR+PHE also characterizes the PAHs of the upper peat horizons as non-pyrogenic. However, in the lower part, the pyrogenic origin of PAHs is confirmed by this coefficient. The FL/PYR ratio has opposite indicators. PAHs in the upper part of the studied peats are pyrogenic, and in the lower horizons, they have a mixed origin. It can be assumed that despite the boundary values of the coefficients, PAHs can be formed as a result of fires. Probably, these ratios should be applied with caution because it is impossible to reliably assert the origin of PAHs.

The ratios FLA/PYR, FLA/(FLA+PYR) and IcdP/(IcdP+BghiP) also serve to assess the formation of PAHs [70,73]. The obtained coefficients characterize the studied PAHs in the majority as pyrogenic. The studied peatlands are characterized by similar patterns of distribution of these diagnostic coefficients. According to the ratios FLA/PYR (<1.4) and IcdP/(IcdP+BghiP; >0.5), the predominance of pyrogenic PAHs in the profile of peats was revealed. However, mainly from the middle of the soil profile (approximately from a depth of 100 cm). According to the literature [74,75,76], these coefficients reflect the burning of grasses, wood and coal, shrubs and other plant residues. The ratio FLA/(FLA+PYR) more accurately conveys the pyrogenic origin of PAHs. The obtained values (>0.5) can be considered evidence as the main source of PAHs of vegetation, coal and wood. Thus, the obtained features of the vertical distribution of PAH concentrations in peat make it possible to more accurately identify the role of paleofires in the formation of the studied peatlands.

## 4. Materials and Methods

### 4.1. Area Description and Soil Sampling

The studies were carried out in the boreal zone of the north-west of the eastern part of Russia. The research area belongs to the Sym-Dubsky Middle Taiga cedar-pine region and is located on the left bank of the Malaya Khoyba River (a tributary of the Yenisei) on the territory of the Sredneenisei station of the V.N. Sukachev Institute of Forest SD RAS (ZOTTO Observatory). The study area is characterized by a sharply continental climate. According to the data of the Bor weather station (the closest to the objects of the study), the average annual air temperature is −3.5 °C, and the average precipitation is 594 mm/g.

The objects of the study were forested peatlands which formed Fibric Histosol (FH). Site FH-1 (60°75′61.11″ N, 89°40′45″ E) is a vast swamp with an area of 160 hectares. Site FH-2 (60°81′31.39″ N, 89°32′99.44″ E) was a sphagnum partially forested swamp with an area of 0.7 hectares. Two soil sections were laid for sampling from peat horizons. In addition, peat columns were taken using an Eijkelkamp soil drill with a sampler diameter of 5 cm and a length of 50 cm.

### 4.2. Botanical Composition

The analysis of the botanical composition and the degree of decomposition of peat was carried out in the Laboratory of Phytocenology and Forest Resource Studies of the Sukachev Institute of Forest, Siberian Branch of the Russian Academy of Sciences (Krasnoyarsk, Russia) according to GOST 28245-89 using a microscope “Leitz Wetzlar” with magnification × 20 and × 40. Stratigraphic diagrams of peat composition were constructed using the software «Korpi» [77]. Atlases were used to identify plant residues in peat [78,79]. Sampling for the analysis of the botanical composition of peat FH-1 was carried out only to a depth of 210 cm.

### 4.3. Charcoal Analysis and ^14^C Dating

The calculation of macroscopic particles of charcoal in peat soils has been out, according to [80]. From the raw column sample, peat was selected with a volume of 1 cm^3^ every 2 cm. Samples were soaked with a 100 cm^3^ 5% NaOCl for 24 h at room temperature to be able to identify black char particles. Then samples for charcoal analysis were gently washed with distilled water through a sieve (0.125 mm), diluted in water in Petri-dishes, and researched for macroscopic charcoal under 40 x stereoscopes. The char particle analysis results were processed in the CharAnalysis program in the R software environment.

Radiocarbon dating of peat samples was carried out at the equipment provided by Shared Research Facilities of the Tomsk Scientific Center SB AS (Tomsk, Russia) by liquid scintillation method using a spectrometer-radiometer Quantulus 1220 (PerkinElmer Life Sciences/Wallac Oy, Turku, Finland). Calibration of the radiocarbon age to calendar age was performed using the CALIB REV–7.10 programs.

### 4.4. Carbon and Nitrogen Analysis

The total C_tot_ and N_tot_ were determined by dry combustion on an EA-1100 analyzer (Carlo Erba, Milano, Italy). Carbon and nitrogen pools were calculated using the formula according to [81]: C_stock_ (kg m^–2^) =0.1×Ctot ×BD ×h, where h is the thickness of the soil layer (cm), C_tot_ is the organic carbon concentration of the soil layer (g kg^−1^), BD is the bulk density of the soil layer (kg m^–3^). A 47.7 cm^3^ drill was used to determine the bulk density of soil horizons.

Water-soluble organic carbon (WSOC) and nitrogen (WSON) were extracted with deionized water (ELGA Lab Water, Lane End, UK) at room temperature at a ratio of 1:50 (soil: water) for mineral horizons and 1:100 for organic and pyrogenic horizons in BIOFIL test tubes with quartz filters (MN, Germany, pore size 0.4 µm). Total carbon (TC) and nitrogen (TN) were assessed using the TOC-VCPN analyzer (Shimadzu, Kyoto, Japan) with a TNM-1 module. The obtained results were recalculated on a dry soil mass basis.

The stable isotope ratios δ^13^C and δ^15^N were determined by an IsoPrime 100 isotope ratio mass-spectrometer (IsoPrime Corporation, Cheadle, UK) and a vario ISOTOPE cube elemental analyzer (Elementar Analysen Systeme GmbH, Hanau, Germany). Stable isotope compositions are reported in delta notation (δ^13^C‰ and δ^15^N‰) relative to Vienna Pee-Dee Belemnite (VPDB) for C, using the international reference materials IAEA-CH-6 (−10.449 ± 0.033‰ VPDB), IAEA-CH-3 (−24.72 ± 0.04‰ VPDB) as standards, and relative to atmospheric N2 for N, using IAEA-N-2 (+20.3 ± 0.2‰ air N2) and USGS-25 (−30.41 ± 0.27‰ air N2) as standards.

### 4.5. 13C-NMR Spectroscopy

The composition of the organic matter was determined by solid-state CP-MAS ^13^C-NMR. Solid-state ^13^C-NMR spectra of the two upper soil horizons were recorded on a 100.53 MHz Bruker Avance III 400WB (Bruker, Ettlingen, Germany) with a rotation frequency of 12.5 kHz, a contact time of 5 ms, and a 2-s recycle delay at the resource center of the research park “Magnetic Resonance Research Methods” of Saint-Petersburg State University, Russia. Before analysis, the samples were treated with 10% hydrofluoric acid to remove paramagnetic iron, according to [82]. Chemical shifts of fractions were determined relative to a tetramethylsilane shift (0 ppm). The contribution of main carbon forms to the total spectral intensity was determined by the integration of the corresponding chemical shift regions according to [83,84]. The total content of aryl C (AR) was calculated as the sum of the signals at 110–165 ppm fields. Signals from alkyl C (AL) were recorded in the 0–110 ppm range. The degree of aromaticity (fa) was determined as the 100% * proportion of total content of aryl C components (110–145 and 145–165 ppm) on total C (excluding the contribution in the range of 165–220 ppm). The hydrophobicity index (Hb) was calculated by the sum (%) of the signals 0–45 and 110–145 ppm.

### 4.6. PAHs Extraction

Chemical analyses were performed in the Chromatography Collective Use Center of the Institute of Biology, Federal Research Center and Komi Research Center of the Ural Branch of the Russian Academy of Sciences. The extraction of PAHs from the soils was made on a Dionex™ ASE™ 350 Accelerated Solvent Extractor (Thermo Fischer Scientific™, Waltham, MA, USA). The extraction was performed three times with a mixture of methylene chloride and acetone (1:1) mixture at 100 °C. The content of polycyclic aromatic hydrocarbons in the concentrates was determined on the basis of US EPA method 8310 (1996a) and certified national standard method of quantitative chemical analysis (PND F 16.1:2.2:2.3:3.62-09. 2009). The relative errors of the determination (*p* = 0.95, ± δ, %) depended on the measurement range and varied within 16–50 for naphthalene (NP), 20–40 for acenaphthene (ACE), 18–40 for fluorene (FL), 20–50 for phenanthrene (PHE), 18–50 for anthracene (ANT), 18–46 for fluoranthene (FLA) and pyrene (PYR), 20–42 for benzo[a]anthracene (BaA), 22–52 for chrysene (CHR), 22–42 for benzo[b]fluoranthene (BbF), 18–48 for benzo[k]fluoranthene (BkF), 18–50 for benzo[a]pyrene (BaP), 20–48 for dibenzo[a,h]anthracene (DahA), and 22–44 for benzo[g,h,i]perylene (BghiP) and indeno[1,2,3-c,d]pyrene (IcdP).

### 4.7. Statistics

Correlation analysis was used to determine the relationship between the obtained data. Correlation coefficients (*r*-Pearson) were calculated using the STATISTICA 10.0 (Stat. Soft Inc, Tusla, OK, USA); differences were considered significant at the significance level *p* < 0.05.

## 5. Conclusions

In our study, a reconstruction of the fire activity of the Western Siberian forest-peatland complexes of the Holocene time period was presented. The studied peatlands have a close history of past fires in the Holocene. It has been established that for 9000 years, the studied peats have been repeatedly confirmed by fires (from 11 to 14 local fires). Two peaks with a maximum content of charcoal particles were characteristic of the Atlantic (~9100–5800 cal. years B.P.) and Subatlantic (~3100 cal. years B.P. to the present) periods of the Holocene. It is shown that the Subboreal period (~5000–2500 cal. years BP) is characterized by a significantly smaller number of fires. There are certainly many reasons for such dynamics of fires (Holocene climate optimum, botanical composition of peat and modern anthropogenic impact). The Holocene climate optimum was a warm period that occurred between about 9000 and 5000 years ago, which contributed to the higher frequency and intensity of fires in the past. A high content of coal particles is characteristic of the lower horizons. It can be assumed that fires in them were a factor that led to the initial waterlogging of the territories. Anthropogenic factors are probably related to the increase in the frequency of fires recorded in recent times (the last 500–1000 years). The effect of fires on soil organic matter is manifested in an increase in carbon reserves in soils and aromatic components in the composition of the SOM. It is suggested that pyrogenesis may affect the increase δ^13^C. There is a significant correlation between the content of PAHs with charcoal particles and with the aromatic structures of SOM in pyrogenic peat layers. Probably, the isolated PAHs are of pyrogenic origin. This is also confirmed by the calculated coefficients of the origin of PAHs. Thus, the content of charcoal particles and the increased content of aromatic fragments of SOM and PAHs in peat horizons can serve as reliable markers of pyrogenesis in peat soils.

## Figures and Tables

**Table 1:** Radiocarbon dating results (^14^C).

Laboratory Sample Number	Sampling Depth, cm	^14^C Age, Years	Age, Cal. Years BP (1σ)
FH-1			
IMCES-^14^C1920	40–60	418 ± 65	497 (400–594)
IMCES-^14^C1930	100–120	1363 ± 65	1367 (1324–1410)
IMCES-^14^C1929	140–160	2040 ± 80	2165 (2146–2183)
IMCES-^14^C1925	180–200	2405 ± 95	2592 (2416–2768)
IMCES-^14^C2108	320–333	6157 ± 130	7137 (6959–7315)
IMCES-^14^C2113	420–432	8370 ± 195	9356 (9105–9606)
FH-2			
IMCES-^14^C1925	60–70	1430 ± 120	1105 (1589–620)
IMCES-^14^C1920	107–108	2915 ± 115	3120 (2960–3280)
IMCES-^14^C1924	120–130	4125 ± 95	4743 (4600–4886)
IMCES-^14^C1890	160–170	4690 ± 130	5512 (5361–5662)
IMCES-^14^C1897	239–240	7535 ± 120	8381 (8267–8495)

**Table 2:** Carbon and nitrogen content in studied Histosols.

Horizon	Depth, cm	C	N	C/N	WSOC	WSON	C/N_WS_	δ ^13^C	δ ^15^N	C_stock_	N_stock_
		g kg^−1^			mg g^−1^			‰		kg m^–2^	
FH-1											
T1	0–20	479 ± 17	6.7 ± 0.7	83	1.36	0.07	23	−27.84	–2.35	1.9	0.03
T2	20–40	493 ± 17	8.3 ± 0.9	69	1.78	0.05	41	−26.57	0.29	2.0	0.03
T3	40–60	460 ± 16	8.4 ± 0.9	64	1.96	0.05	43	−27.00	1.63	7.4	0.13
T4	60–80	453 ± 16	6.7 ± 0.7	79	2.15	0.07	35	−26.73	–0.08	7.2	0.11
T5	80–100	477 ± 17	8.3 ± 0.9	67	1.21	0.04	38	−27.41	0.05	7.6	0.13
T6	100–120	500 ± 18	9.8 ± 1.1	60	1.65	0.06	31	−27.47	–0.34	8.0	0.16
T7	120–140	456 ± 16	5.6 ± 1.1	95	1.55	0.06	31	−26.69	–1.89	7.3	0.09
T8	140–160	472 ± 16	7.8 ± 0.9	71	1.41	0.05	30	−27.07	–1.05	7.6	0.12
T9	160–180	463 ± 16	7.2 ± 0.8	75	1.61	0.07	27	−26.74	–1.62	7.4	0.12
T10	180–200	480 ± 17	8.0 ± 0.9	70	1.73	0.08	26	−26.66	–1.82	7.7	0.13
T11	200–220	512 ± 18	26.3 ± 2.9	19	–	–	–	−25.54	–1.17	8.2	0.42
T12	220–240	503 ± 18	35.5 ± 4.5	14	–	–	–	−26.53	–0.91	8.0	0.57
T13	240–260	551 ± 19	22.8 ± 2.4	24	–	–	–	−28.04	0.70	8.8	0.36
T14	260–280	546 ± 19	23.7 ± 2,5	23	–	–	–	−28.03	0.39	8.7	0.38
T15	280–300	541 ± 19	22.2 ± 2,4	24	–	–	–	−27.98	0.22	8.7	0.36
T16	300–330	563 ± 20	17.7 ± 1.8	32	–	–	–	−28.24	0.53	9.0	0.28
T17	330–360	537 ± 19	20.4 ± 2.3	26	–	–	–	−27.93	0.19	8.6	0.33
T18	360–380	525 ± 18	17.6 ± 1.8	30	–	–	–	−26.95	–1.06	8.4	0.28
T19	380–400	531 ± 19	19.5 ± 1.9	27	–	–	–	−27.49	0.27	8.5	0.31
T20	400–420	544 ± 19	20.2 ± 2.3	27	–	–	–	−28.21	1.14	8.7	0.32
										149.7	4.7
FH-2											
T1	0–20	478 ± 17	7.1 ± 0.8	79	1.18	0.03	42	−27.50	–4.60	1.9	0.03
T2	20–40	498 ± 17	15.5 ± 1.7	37	1.46	0.07	26	−26.82	1.00	14.9	0.47
T3	40–60	507 ± 18	14.4 ± 1.6	41	0.99	0.04	27	−27.14	–0.37	17.2	0.49
T4	60–80	519 ± 18	14.1 ± 1.6	43	1.32	0.06	27	−27.19	–1.48	16.6	0.45
T5	80–100	518 ± 18	17.2 ± 1.9	35	1.28	0.06	24	−26.88	–0.57	18.6	0.62
T6	100–120	525 ± 18	20.6 ± 2.3	30	1.35	0.08	20	−26.59	–0.67	16.8	0.66
T7	120–140	528 ± 18	26.6 ± 2.9	23	1.63	0.17	11	−25.31	–0.86	15.8	0.80
T8	140–160	528 ± 18	28 ± 3	22	1.21	0.10	15	−24.79	–1.26	16.9	0.90
T9	160–180	523 ± 18	31 ± 3	20	1.23	0.11	13	−24.07	–0.98	16.7	0.99
T10	180–200	515 ± 18	27 ± 3	22	1.11	0.10	12	−26.37	–1.04	16.5	0.86
T11	200–230	535 ± 19	27 ± 3	23	0.90	0.13	8	−25.14	–1.37	25.7	1.30
T12	230–235	533 ± 19	20.6 ± 2.3	30	0.60	0.08	9	−27.72	0.12	4.3	0.16
Tpyr	235–240	258 ± 26	8.8 ± 1.0	34	0.32	0.03	13	−28.04	1.51	2.1	0.07
G	240–260	27 ± 4	1.31 ± 0.26	24	0.09	0.02	7	nd	nd	nd	nd
										184.1	7.8

Note: ± –analytical error. WSOC–water-soluble organic carbon, WSON–water-soluble organic nitrogen, nd – did not determine. To calculate stocks from a depth of 140 cm and deeper, median values of the density of peat horizons were used.

**Table 3:** Percentage distribution (%) of signal intensity between selected chemical shift regions (ppm) of ^13^CPMAS NMR spectra of bulk soil.

Depth, cm	Alkyl C	O-Alkyl C			Aryl C		Carboxyl C/ Amide/Easter		Alkyl/O,N-alkyl		Aromaticity (fa)
	C_Alk-H_	C_CH3-O_	C_Alk-O_	C_O-Alk-O_	C_Ar-H(C)_	C_Ar-O,N_	C_COOH(R)_	C_C=0_			
	0–45	45–60	60–95	95–110	110–145	145–165	165–185	185–220			
FH-1											
0–20	16.6	6.8	41.4	12.1	12.8	5.8	3.8	0.7	0.3	18.6	
20–40	25.9	7.4	35.9	10.3	10.8	5.1	4.4	0.3	0.5	15.9	
40–60	17.9	6.2	47.5	11.3	9.7	3.4	4.0	0.0	0.3	13.2	
60–80	15.4	6.3	51.2	11.4	9.4	3.3	3.1	0.0	0.2	12.6	
80–100	20.9	6.5	46.0	10.4	10.2	3.0	3.1	0.0	0.3	13.2	
100–120	24.5	6.0	39.2	9.9	12.4	4.4	3.7	0.0	0.4	16.7	
120–140	17.5	5.2	46.2	11.8	11.4	4.4	3.6	0.0	0.3	15.7	
140–160	16.8	5.6	46.7	11.2	11.9	4.1	3.5	0.0	0.3	16.0	
160–180	18.2	6.5	44.0	10.8	13.3	4.6	2.7	0.0	0.3	17.8	
180–200	19.9	6.1	42.8	10.6	12.8	4.4	3.3	0.0	0.3	17.3	
200–220	21.6	8.9	32.5	8.4	14.4	5.7	6.6	1.9	0.4	20.1	
220–240	23.9	9.3	29.0	7.7	14.2	5.6	8.0	2.3	0.5	19.8	
240–260	32.7	8.3	23.3	6.0	14.8	6.0	6.5	2.4	0.9	20.8	
260–280	30.5	8.7	24.6	6.4	15.7	6.0	5.9	2.2	0.8	21.6	
280–300	26.6	8.6	25.8	7.2	17.1	6.9	5.7	2.1	0.6	24.0	
300–330	28.5	9.5	21.3	6.4	19.4	7.8	5.0	2.1	0.8	27.2	
330–360	28.1	8.2	25.0	7.0	17.1	6.8	5.8	1.9	0.7	23.9	
360–380	24.7	7.1	29.4	8.0	16.3	7.0	5.5	2.2	0.6	23.2	
380–400	24.4	7.6	26.2	7.7	18.1	7.6	5.9	2.5	0.6	25.7	
400–420	27.3	8.2	23.2	7.3	18.7	7.8	5.5	2.0	0.7	26.5	
FH-2											
0–20	16.2	6.9	41.4	12.2	13.5	6.2	3.5	0.0	0.3	19.7	
20–40	24.0	8.0	37.4	9.8	12.1	4.2	4.5	0.0	0.4	16.3	
40–60	26.2	8.0	33.7	9.7	13.8	4.6	3.9	0.0	0.5	18.4	
60–80	26.8	7.8	31.9	9.4	15.0	5.4	3.8	0.0	0.5	20.3	
80–100	25.7	8.2	30.5	9.0	16.2	6.0	4.4	0.0	0.5	22.2	
100–120	26.5	8.9	29.2	8.7	16.7	5.4	4.7	0.0	0.6	22.1	
120–140	27.7	9.9	27.4	7.9	16.9	5.3	4.9	0.0	0.6	22.3	
140–160	25.0	9.6	30.6	8.7	15.7	4.8	5.6	0.0	0.5	20.5	
160–180	25.8	10.2	28.2	8.4	16.6	5.3	5.6	0.0	0.6	21.9	
180–200	27.2	9.6	26.9	8.4	17.0	5.8	5.0	0.0	0.6	22.8	
200–230	30.1	11.5	25.4	8.2	16.9	4.6	3.3	0.0	0.7	21.5	
230–235	32.6	9.9	20.8	7.1	19.5	6.0	4.2	0.0	0.9	25.5	
235–240	37.6	6.8	12.6	5.3	26.4	6.3	4.9	0.0	1.5	32.7	

## Data Availability

Not applicable.

## Appendix A

**Table A1:** Content of PAHs in soils, ng g^−1^.

Depth, cm	2-Rings			3-Ring		4-Ring				5-Ring				6-Ring		∑	∑LP	∑HP
	NP	ACE	FL	PHE	ANT	FLA	PYR	BaA	CHR	BbF	BkF	BaP	DahA	BghiP	IcdP			
FH-1																		
0–20	43	0	6	39	2	8	4	1	3	3	1	1	1	59	0	170	106	64
20–40	67	0	10	59	2	12	3	1	6	6	4	2	1	0	0	173	160	13
40–60	50	0	8	59	2	5	1	0	1	3	1	0	0	0	0	130	126	4
60–80	41	0	9	46	4	7	2	0	3	3	1	2	1	3	0	123	113	10
80–100	61	0	6	34	1	3	2	0	2	2	0	0	4	2	0	117	109	8
100–120	64	0	9	43	2	3	5	0	2	3	0	0	1	2	0	133	127	7
120–140	42	0	7	39	1	3	4	0	2	0	0	0	2	0	0	101	98	3
140–160	60	0	6	32	1	3	2	0	2	2	0	0	1	0	0	109	106	3
160–180	50	0	5	35	1	3	2	0	2	0	0	0	0	0		99	99	0
180–200	42	0	6	34	2	4	0	1	2	0	0	0	13	11	56	171	90	81
200–220	140	0	35	190	10	26	21	n.d.	8	11	n.d.	1	19	7	28	496	431	65
220–240	110	0	32	170	10	37	63	n.d.	15	13	n.d.	2	18	15	160	646	438	209
240–260	340	11	80	490	27	49	34	n.d.	20	n.d.	n.d.	3	160	170	760	2142	1049	1093
260–280	230	10	63	350	22	63	45	n.d.	17	12	1	2	12	24	200	1051	799	252
280–300	200	9	57	330	20	66	70	n.d.	n.d.	n.d.	n.d.	1	7	n.d.	12	772	752	20
300–330	390	13	91	530	27	100	90	10	30	11	2	5	n.d.	9	14	1323	1282	41
330–360	200	n.d.	47	210	12	39	47	n.d.	21	9	1	4	590	510	1020	2710	576	2134
360–380	70	n.d.	21	150	8	28	46	n.d.	15	140	3	2	58	37	810	1390	338	1051
380–400	260	n.d.	50	260	14	30	56	n.d.	21	16	2	3	210	490	1700	3114	692	2422
400–420	320	11	86	400	27	54	48	n.d.	20	100	42	2	510	750	2000	4370	966	3404
FH-2																		
0–20	33	0	4	22	1	8	1	0	1	0	1	1	0	0	0	71	70	2
20–40	63	0	6	30	1	2	1	0	2	0	0	0	0	5	0	111	106	6
40–60	70	0	4	33	1	3	2	0	2	0	0	0	16	9	30	169	114	55
60–80	74	0	5	35	2	3	2	0	5	6	1	1	12	0	74	221	128	94
80–100	83	0	5	23	1	1	2	0	2	0	1	7	57	0	318	501	118	383
100–120	77	0	8	43	2	6	3	0	2	0	0	0	41	0	182	364	141	223
120–140	55	0	8	42	2	7	7	0	3	3	1	1	21	0	14	164	123	41
140–160	94	0	10	57	5	5	6	0	0	2	1	1	84	0	391	656	178	479
160–180	144	0	11	65	4	9	7	0	0	23	2	6	30	0	558	858	239	619
180–200	627	0	10	60	16	12	7	0	0	51	3	19	126	0	1043	1974	733	1241
200–230	130	0	10	62	4	10	9	0	0	18	3	3	15	0	865	1127	224	903
230–235	545	0	9	64	5	8	2	0	28	38	1	13	18	0	638	1370	662	708
235–240	192	0	26	99	7	16	17	2	14	2	1	3	5	0	0	384	373	11

Note: n.d.–not detected (for ACE <6, FL <6, FLA <20, PYR <20, BaA <6, CHR <3, BbF <6, BkF <1, BaP <1, DahA <6, BghiP <6, IcdP <6). NP–naphthalene, ACE–acenaphthene, FL–fluorene, PHE–phenanthrene, ANT–anthracene, FLA–fluoranthene, PYR–pyrene, BaA–benzo[a]anthracene, CHR–chrysene, BbF–benzo[b]fluoranthene, BkF–benzo[k]fluoranthene, BaP–benzo[a]pyrene, DahA–dibenzo[a,h]anthracene, BghiP–benzo[g,h,i]perylene, IcdP–indeno [1,2,3-c,d]pyrene.

**Table A2:** The diagnostic ratios of PAHs.

Depth, cm	PHE/ANT	ANT/178	ANT/(ANT+PHE)	FLA/PYR	FLA/(FLA+PYR)	BaA/(BaA+CHR)	IcdP/(IcdP+BghiP)	FL/PYR	(PYR+FLA)/(CHR+PHE)	(PYR+BaP)/CHR+PHE)
	<10	>0.1	>0.1	<1.4	>0.5	>0.35	>0.5	>1	>0.5	>0.1
FH-1										
0–20	20	0.0	0.0	2.1	0.7 **	0.28	0.0	2 **	0.3	0.1 *
20–40	24	0.0	0.0	4.2	0.8 **	0.16	0.0	3 **	0.2	0.1 *
40–60	25	0.0	0.0	3.3	0.8 **	0.15	0.0	6 **	0.1	0.0
60–80	13	0.0	0.1 *	3.3	0.8 **	0.16	0.0	4 **	0.2	0.1 *
80–100	27	0.0	0.0	1.6	0.6 **	0.15	0.0	3 **	0.1	0.1 *
100–120	25	0.0	0.0	0.5 **	0.3	0.11	0.0	2 **	0.2	0.1 *
120–140	28	0.0	0.0	0.7 **	0.4	0.00	0.0	2 **	0.2	0.1 *
140–160	27	0.0	0.0	1.6	0.6 **	0.00	0.0	3 **	0.1	0.1 *
160–180	31	0.0	0.0	1.2 **	0.6 **	0.00	0.0	2 **	0.2	0.1 *
180–200	22	0.0	0.0	0.0	1.0 **	0.24	0.8 **	0	0.1	0.0
200–220	18	0.1 *	0.1 *	1.2 **	0.5 **	0.00	0.8 **	2 **	0.2	0.1 *
220–240	17	0.1 *	0.1 *	0.6 **	0.4	0.00	0.9 **	1 *	0.5	0.4 **
240–260	18	0.1 *	0.1 *	1.4	0.6 **	0.00	0.8 **	2 **	0.2	0.1 *
260–280	16	0.1 *	0.1 *	1.4	0.6 **	0.00	0.9 **	1 *	0.3	0.1 *
280–300	16	0.1 *	0.1 *	0.9 **	0.5 **	0.00	1.0 **	1 *	0.4	0.2 **
300–330	19	0.2 **	0.0	1.1 **	0.5 **	0.24	0.6 **	1 *	0.3	0.2 **
330–360	17	0.1 *	0.1 *	0.8 **	0.5 **	0.00	0.7 **	1 *	0.4	0.2 **
360–380	19	0.0	0.0	0.6 **	0.4	0.00	1.0 **	0	0.4	0.3 **
380–400	18	0.1 *	0.1 *	0.5 **	0.4	0.00	0.8 **	1 *	0.3	0.2 **
400–420	15	0.2 **	0.1 *	1.1 **	0.5 **	0.00	0.7 **	2 **	0.2	0.1 *
FH-2										
0–20	28	0.0	0.0	8.3	0.9 **	0.33	0.0	4 **	0.4	0.1 *
20–40	21	0.0	0.0	1.7	0.6 **	0.00	0.0	5 **	0.1	0.1 *
40–60	56	0.0	0.0	1.7	0.6 **	0.00	0.8 **	2 **	0.1	0.1 *
60–80	18	0.0	0.1 *	1.5	0.6 **	0.05	1.0 **	2 **	0.1	0.1 *
80–100	21	0.0	0.0	0.5 **	0.3 **	0.00	1.0 **	2 **	0.1	0.4 **
100–120	25	0.0	0.0	1.9	0.7 **	0.13	1.0 **	3 **	0.2	0.1 *
120–140	21	0.0	0.0	1.0 **	0.5 **	0.12	1.0 **	1 *	0.3	0.2 **
140–160	12	0.0	0.1 *	0.9 **	0.5 **	1.00 **	1.0 **	2 **	0.2	0.1 *
160–180	17	0.0	0.1 *	1.2 **	0.5 **	0.00	1.0 **	1 *	0.3	0.2 **
180–200	4 **	0.1 *	0.2 **	1.7 **	0.6 **	0.00	1.0 **	1 *	0.3	0.4 **
200–230	16	0.0	0.1 *	1.1 **	0.5 **	1.00 **	1.0 **	1 *	0.3	0.2 **
230–235	12	0.0	0.1 *	3.4	0.8 **	0.00	1.0 **	4 **	0.1	0.2 **
235–240	14	0.0	0.1 *	1.0 **	0.5 **	0.15	0.0	2 **	0.3	0.2 **
–pyrogenic origin –boundary value										
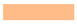
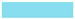

Note: *–boundary value, **–pyrogenic origin, notation as in Figure 4.
